# Thrombin: An Approach to Developing a Higher-Order Reference Material and Reference Measurement Procedure for Substance Identity, Amount, and Biological Activities

**DOI:** 10.6028/jres.125.021

**Published:** 2020-07-29

**Authors:** Craig M. Jackson, Peter Esnouf, David L. Duewer

**Affiliations:** 1 5931 Seacrest View Road San Diego, CA 92121-4355 USA; 2 7 Stonesfield Road Combe, Witney, OX29 8PF, UK; 3 National Institute of Standards and Technology, Gaithersburg, MD 20899, USA

**Keywords:** blood clotting, clotting activity, exosite, fibrinogen, fibrinopeptide, heparin, peptide p-nitroanilide, thrombin, α-thrombin, β-thrombin, γ-thrombin

Thrombin, the proteolytic enzyme that catalyzes the transformation of soluble fibrinogen to the polymerized fibrin clot, participates in multiple reactions in blood coagulation in addition to the clotting reaction. Although reference materials have existed for many years, structural characterization and measurement of biological activity have never been sufficient to permit claims of clear metrological traceability for the thrombin preparations. Our current state-of-the-art methods for protein characterization and determination of the catalytic properties of thrombin now make it practical to develop and characterize a metrologically acceptable reference material and reference measurement procedure for thrombin. Specifically, α-thrombin, the biologically produced protease formed during prothrombin activation, is readily available and has been extensively characterized. Dependences of thrombin proteolytic and peptide hydrolytic activities on a variety of substrates, pH, specific ions, and temperature are established, although variability remains for the kinetic parameters that describe thrombin enzymatic action. The roles of specific areas on the surface of the thrombin molecule (exosites) in substrate recognition and catalytic efficiency are described and characterized. It is opportune to develop reference materials of high metrological order and technical feasibility. In this article, we review the properties of α-thrombin important for its preparation and suggest an approach suitable for producing a reference material and a reference measurement procedure that is sensitive to thrombin’s catalytic competency on a variety of substrates.

## 1 Introduction

Understanding the challenge of developing a reference material and reference measurement procedures for both substance amount and quantification of biological activity for thrombin is a prerequisite to an appropriate reference system for this biological material. More specifically, a system suitable for the intended uses of the material and procedure must include clear definition of the identity of the substance as determined by its structure and its structure-determined biological activities.

Identification of the structure-function relationships that are documented for thrombin is facilitated by a brief description of the biological system in which thrombin participates and in which it is produced, *i.e*., a description of the biological context of thrombin. Because thrombin, initially called “fibrin ferment,” was first postulated to be the agent causing the transformation of flowing blood into a gel in 1872 [1], an account of some of the legacy of prior approaches to developing reference materials for thrombin biological activity assessment is helpful. This discussion is intended to enable the previously encountered impediments to a metrology-based reference system for thrombin to be avoided.

**Fig. 1:**
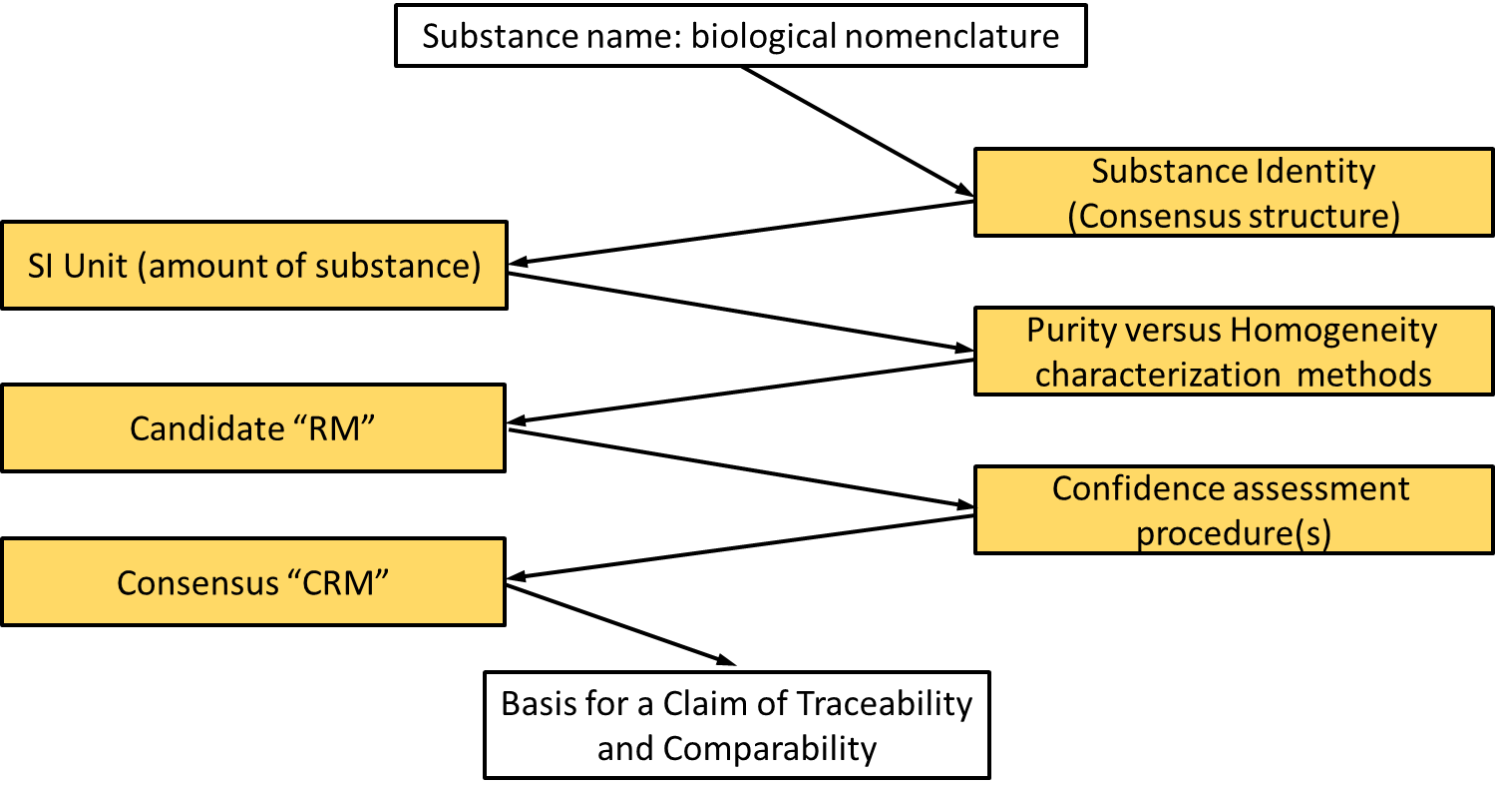
A modified traceability path for macromolecules of biological origin. Consensus on the structure that will define the macromolecule is required because of the inherent heterogeneity that exists because of genetic sequence and posttranslational polymorphisms. Historical names are maintained for recognitions of the substance, but the agreed amino sequence, after acceptance, becomes the defining structure. An “RM” is a reference material in which the measurand is known to be fit-or-purpose homogenous and stable but for which the true value of the measurand has not been sufficiently well-established. A “CRM” is a certified RM for which an interval containing the true value of the measurand has been established with a stated level of confidence.

The production of thrombin, as a substance rather than an “activity” capable of initiating clot formation, was first achieved in the late 1930s [2]. A commercial product employed to stop bleeding, “Thrombin, Topical,” was developed by Parke, Davis and Company and used in both clinical and research applications. Identification of other coagulation factor activities in “Thrombin, Topical” [3, 4] and its somewhat unsatisfactory physical appearance (turbid, gray, and with evident particulates) occurred as technology advanced. The development of large-scale purification of thrombin from Cohn fraction III [5] led to better and, in many cases, highly active and apparently homogeneous preparations that were stable and widely usable as a reference material for measuring thrombin activity [6]. Continuing until today, thrombin “standards,” sponsored by the World Health Organization (WHO) and U.S. National Institutes of Health (NIH), provide the basis for measurement of thrombin biological activity [7]. However, characterization of these thrombin reference materials has been limited and not focused on substance homogeneity, amount, or biological activity as necessarily linked properties. The goal of this document is to outline an approach that can meet metrological criteria for traceability and serve as a guide to developing a higher-order reference material for both substance amount and biological activity; the proposed approach is illustrated as a traceability path in Fig. 1.

## 2 Thrombin

A voluminous body of literature for thrombin exists; more than 48,000 publications are listed in Medline, and about 450 crystallographic structures are reported in the Protein Data Bank [8], wherein many structures are of thrombin-drug and thrombin-inhibitor complexes. If a defensible consensus structure for thrombin is to be decided, candidates must be comprehensively evaluated with respect to the structural information that exists in the literature and the functional consequences of differences in structure. Thus, a focused, although necessarily limited, review of the structural and functional properties of thrombin needs to be made prior to proposing a reference structure for a highest-order reference material. An inappropriate choice of sequence could have unintended adverse consequences by introducing bias in the proposed reference measurement procedure. Inappropriate in this context is taken to mean that sequence variants (mutations) known to affect structure (tertiary or quaternary) and/or any of thrombin’s biological function should be excluded.

Recombinant thrombin can be produced based on the consensus sequence [9, 10, 11], but it would be expensive compared to that from human plasma. Fortunately, naturally occurring mutations with functional impairment are rare in thrombin, thus making them unlikely to affect the protein isolated from human plasma. Residues within the thrombin sequence have been identified as functionally important from study of those rare mutations and from recombinant thrombins in which residues have been replaced by site-directed mutagenesis. Screening for the presence of significant amounts of such undesirable residues in the sequence can be done, and any pools of plasma with a high prevalence of them can be excluded or reduced to inconsequential prevalence using contemporary technologies such as protein mass spectroscopy (MS). Inconsequential can be interpreted to mean within the measurement uncertainty for the analytical procedure.

### 2.1 Substance Identity, Name versus Biochemical Entity

As the first step to developing a thrombin reference material that is metrologically traceable, the explicit primary structure that will identify the substance “thrombin” (specifically, α-thrombin^1^1 Unless stated otherwise, thrombin implies α‑thrombin, the physiologically relevant and fully competent proteolytic enzyme that converts fibrinogen to fibrin.) must be decided. Because of the inherent, although limited, amino acid sequence heterogeneity of proteins of biological origin, the amino acid sequence for “the reference thrombin” must be decided by consensus. A proposed sequence will be suggested that addresses the heterogeneity in a way that is both practical and that identifies the preparation of thrombin explicitly but allows for new discoveries of structure-function relationships for thrombin to be considered and included in the proposed reference system. If possible (economically), the actual sequence of the protein preparation with heterogeneities in sequence could be determined.

The purity of the thrombin preparation must be established. It is worth noting, however, that purity of preparations of macromolecules, and the evidence used to claim minimal heterogeneity, was first recognized as the result of a process of exclusion, rather than by properties that can be used with simple substances. This was presciently stated in 1940 [12]: “…purity is a concept that has no meaning except with reference to the methods and assumptions used in studying the substance….” More explicitly, the fidelity of the material chosen to represent the sequence selected to identify the substance thrombin depends on the sensitivity and selectivity of the analytical methods employed to detect contaminants (impurities) and the number of different properties characterized. By this approach, metrological confidence can be established, and the acceptability of the resulting thrombin preparation as the highest-order reference material can be judged suitable for its intended uses.

### 2.2 Biochemical and Structural Context

Thrombin is a member of the serine protease family (EC 3.21.5), the group of enzymes in which chymotrypsin and trypsin are the classical reference enzymes [13]. Although initially categorized based on the “charge-relay” mechanism of action, amino acid sequences showed that trypsin and chymotrypsin are structurally homologous proteins [14]. After completion of the amino acid sequence for bovine thrombin, it was evident that thrombin is homologous in the underlying scaffold to these proteases. Notable differences were observed; in particular, insertions were found in the thrombin sequence. These insertions were commonly at locations predicted to be on the surface of the thrombin molecule [15, 16]. When the three-dimensional structure of thrombin was reported, it was clear that the inserted regions were in fact on the surface of the thrombin molecule, offering plausible explanations for differences in specificity toward protein substrates between thrombin and trypsin.

**Fig. 2:**
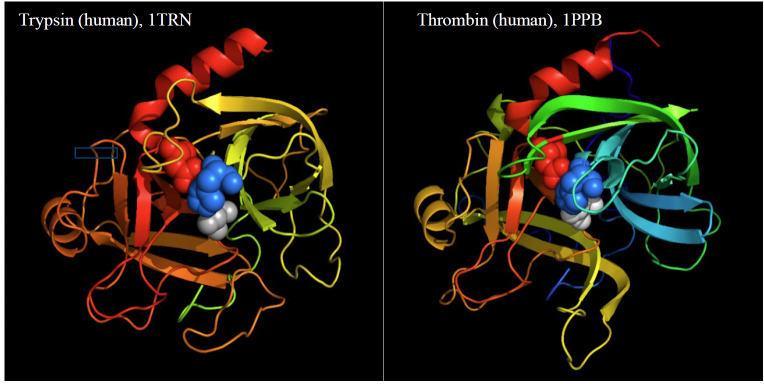
Trypsin versus thrombin— related proteases with property-related differences. Comparison of thrombin with the more widely recognized protease trypsin facilitates identification of the functional differences in thrombin that result from the insertions of amino acid sequences that create regulatory sites on thrombin. Trypsin can proteolytically cleave all arginyl and lysyl peptide bonds in proteins; thrombin is primarily directed to arginyl residues on its protein substrates, partly determined by accessibility and partly by the expanded active site region in thrombin. Trypsin structure 1TRN is from Ref. [21]; thrombin structure 1PPB is from Ref. [22]. All molecular structures in this report were created with PyMOL, an open-source molecular visualization system (https://en.wikipedia.org/wiki/PyMOL).

Prior to the three-dimensional structure for thrombin, many investigators compared thrombin with trypsin, focusing on the notable similarities [17]; however, other investigators focused on the differences between thrombin and trypsin and began investigating the possible functional significance of these additional surface residues. An early proposal was that the surface residues might create exosites, *i.e*., sites distinct and remote from the catalytic or active site of thrombin [18, 19]. Exosites quickly came to be the focus of investigations directed to describing and differentiating among the interactions between thrombin and its many protein substrates [20]. Ribbon diagrams for trypsin and thrombin (see Fig. 2) illustrate both the similarities and differences between these two proteolytic enzymes.

#### 2.3 Primary, Secondary, and Tertiary Structures

**Fig. 3:**
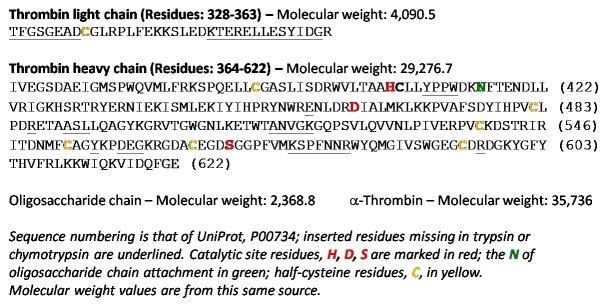
Amino acid sequence of thrombin, showing catalytic triad and inserted sequences. The inserted sequences suggested in Refs. [16, 26, 27] are underlined to illustrate the structural differences from trypsin that are responsible for the specificity exhibited by thrombin for the multiple protein substrates on which it acts. Catalytic site residues, *H*, *D*, *S*, are marked in red; the *N* of the oligosaccharide chain attachment is in green; half-cysteine residues, *C*, are in yellow. The sequence and molecular weights are from UniProt P000734.

**Fig. 4:**
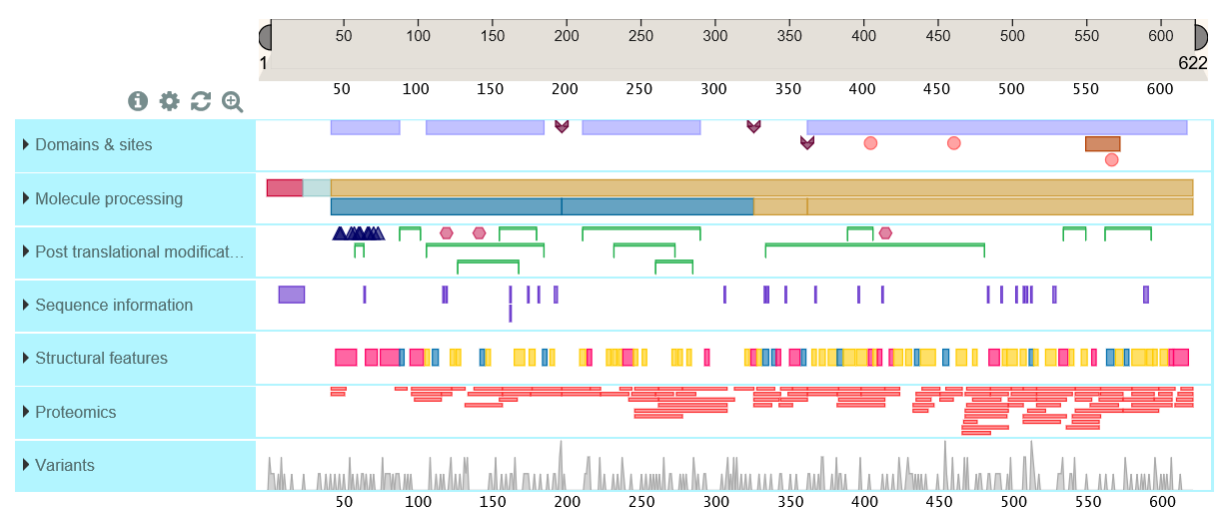
UniProtKB, a most useful source for structural information for proteins. UniProtKB (KnowledgeBase) is a curated, hierarchically organized database that provides extensive structural and brief functional information for proteins.

It is proposed that the “defining” primary structure of thrombin given in the UniProt database [23], a highly curated protein structure database, be established as the consensus amino acid sequence for human α-thrombin (Fig. 3). The sequence is derived from both protein and complementary deoxyribonucleic acid (cDNA) sequences [24, 25]. Subsequent differences in residues are found in X-ray crystallographic structures, documented in the Protein Data Bank files [8]. Tables of sequence conflicts and naturally occurring variants are given in the UniProt database, http://www.uniprot.org/uniprot/P00734#sequences. Limited numbers of residues with sequence conflicts and residues shown by site-directed mutagenesis to be important are shown in Fig. 4.

**Fig. 5:**
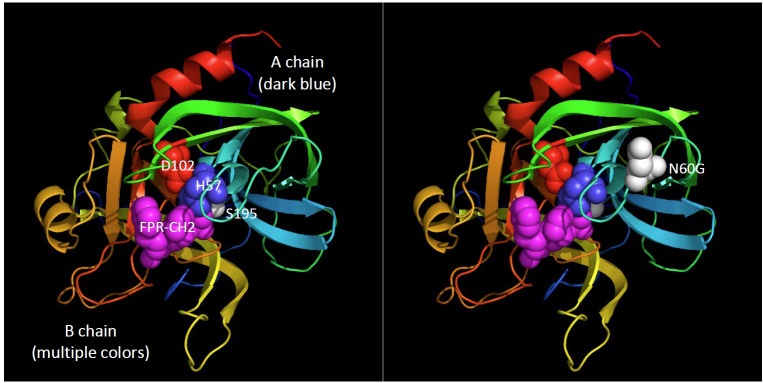
Thrombin, showing the catalytic triad and the location of the single oligosaccharide chain. The catalytic triad, serine 195 (S195), histidine 57 (H57), and aspartate 102 (D102), are the residues that are directly responsible for the proteolytic activity of serine proteases. An oligosaccharide chain is attached to asparagine, N60G. (Numbering is that first reported for chymotrypsin to facilitate identification on three-dimensional structures because crystallographers employ this system. The Appendix cross-references the several thrombin numbering systems used in the literature.) The Phe-Pro-Arg-CH_2_ residue (FPR-CH_2_) is a covalent inhibitor that has reacted with H57 to provide a proteolytically inactive thrombin that can be crystallized. This sequence is from UniProt P000734.

Structural features revealed in the three-dimensional structure of thrombin underlie many of the functional attributes that confer the specificity that thrombin exhibits toward protein substrates. Figure 5 shows an annotated three-dimensional structure derived from the Protein Databank Entry, 1PPB, the first reported structure for human thrombin [22, 28].

### 2.4 Exosites—Distinguishing Structural Features of Thrombin and Other Coagulation Proteases

Exosites (protein ligand binding sites), composed of residues identifiable at or near the surface of the thrombin molecule, provide for the special interactions between thrombin and protein substrates [20]. Ligands, when bound at these sites, alter the specificity and catalytic efficiency of thrombin. The first clear indication of the importance of an exosite arose from the interaction of thrombin with the polypeptide hirudin (65 amino acid residues) from the leach *Hirudo medicinalis* [29, 30]. Hirudin has been studied extensively because of its ability to prevent blood from clotting [31, 32]. Hirudin’s very tight binding to thrombin suggested interactions beyond the active site; this was confirmed by the publication of the three-dimensional structure of the thrombin-hirudin complex [33, 34] and direct binding measurements [31, 35, 36]. Portions of the hirudin molecule block access to the active site; however, most of the hirudin is folded around a groove in the surface of thrombin away from the active site [37, 38]. Interaction between the hirudin “tail” and the residues away from the active site operationally defines the exosite, subsequently designated exosite 1. Several other proteins bind to thrombin at exosite 1, each with distinguishable effects on thrombin’s specificity [39, 40, 42].

Evidence that heparin formed a ternary complex between thrombin and the inhibitor antithrombin during the heparin-catalyzed inactivation of thrombin by antithrombin implied another exosite on thrombin [41, 42]. This interaction site on thrombin, now known as “exosite 2,” is not limited to interacting with heparin and other glycosaminoglycans, but proteins as well. One interaction with exosite 2 involves binding of one of the activation fragments (prothrombin fragment 2) [43]. This fragment and the complete “pro-half” of prothrombin are produced during prothrombin conversion to thrombin. This interaction is involved in the prothrombinase-catalyzed activation of prothrombin [44, 45] and has been observed crystallographically [75] to alter thrombin action on a model protein substrate [46]. The structural integrity and the absence of substances that interact with thrombin exosites are important considerations regarding the purity of a thrombin reference material.

**Fig. 6:**
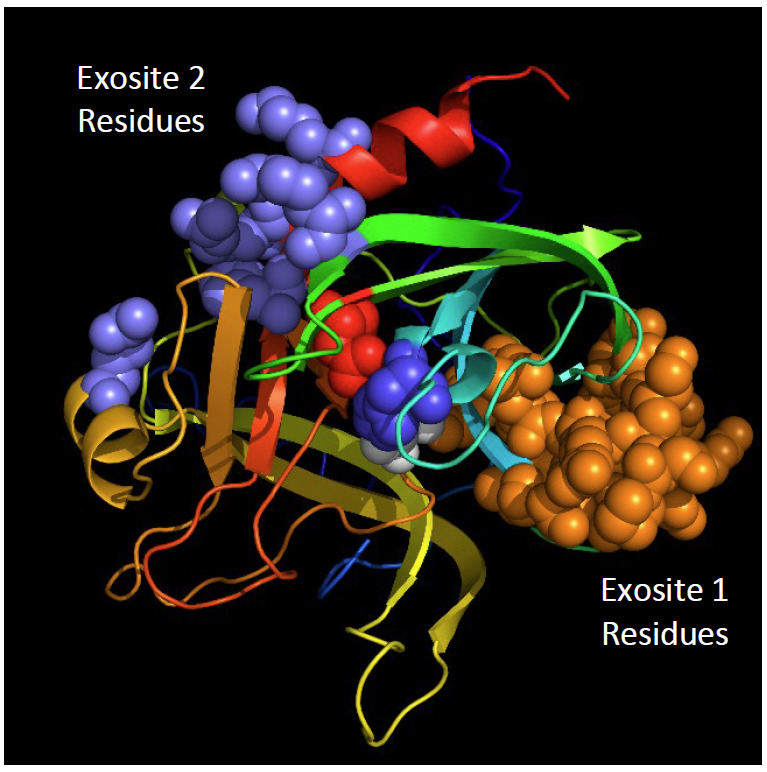
Exosites on the thrombin surface—determinants of thrombin specificity. Exosites 1 and 2 are created from the inserted amino acid sequences (Figs. 2 and 3) and are responsible for the recognition sites for thrombin’s many macromolecular substrates, its inhibitors, and the glycosaminoglycan, heparin. Residues implicated in the exosites are from Ref. [47].

**Fig. 7:**
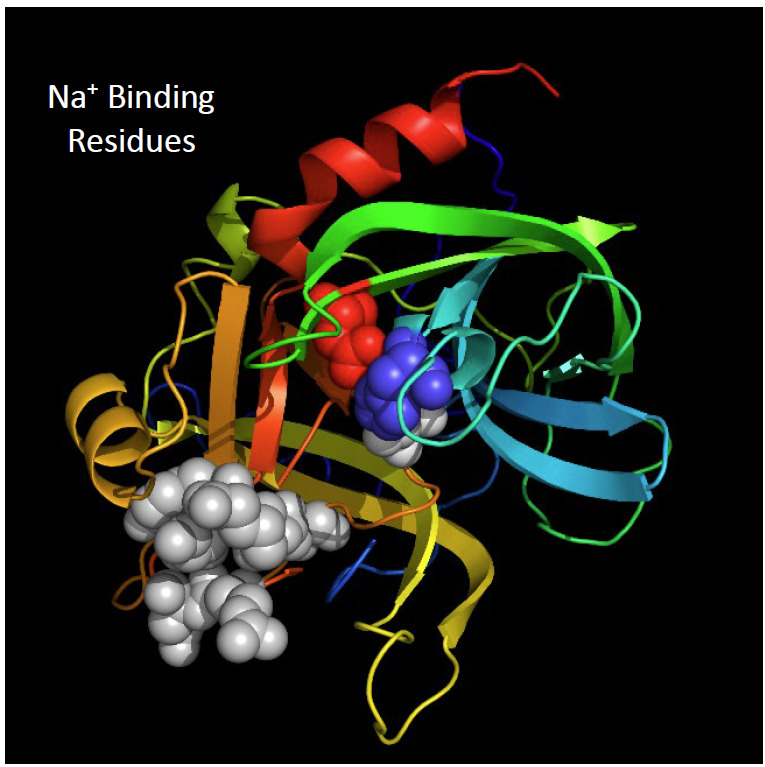
The Na^+^ binding site of thrombin. Two conformers of thrombin exist, a “fast form,” which is the most efficient conformation for cleavage of the fibrinopeptides from fibrinogen, and a “slow form,” which is the most efficient conformation for activation of protein C when in complex with the cofactor protein thrombomodulin. The “fast form” favors clotting; the “slow form” participates in reactions that shut off thrombin formation. The Na^+^ site is fully occupied in the “fast form”; it is unoccupied in the “slow form.” Residue identification is from Refs. [81, 86].

**Fig. 8:**
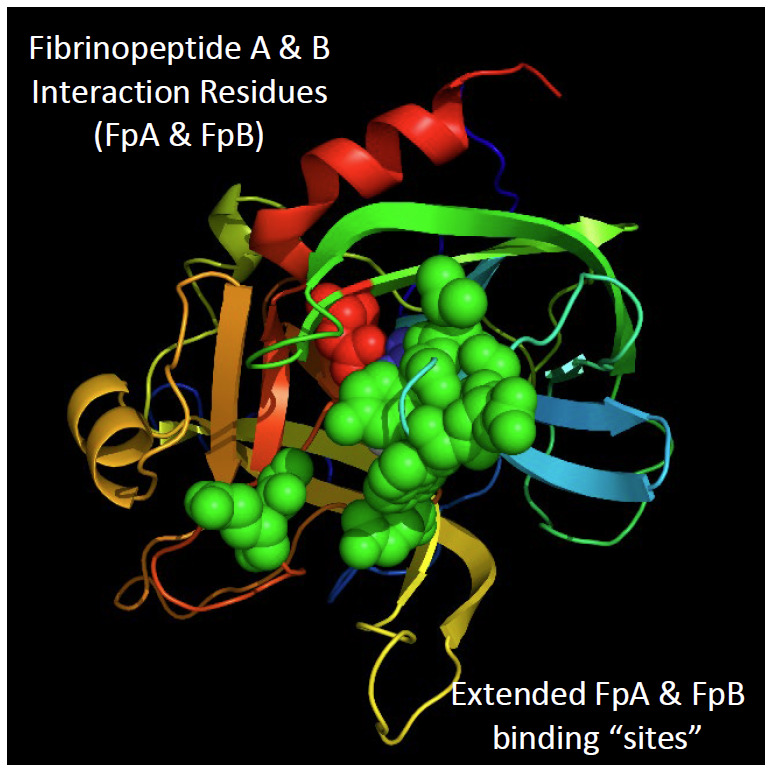
The expanded active site of thrombin. Restriction of thrombin action on its protein substrates is determined by its expanded active site. Shown here are residues related to binding the fibrinopeptides as determined by crystallography of a fibrinopeptide-thrombin complex [51, 54, 55].

Exosites and amino acid residues in portions of the thrombin polypeptide chain adjacent to the active site have been investigated by site-directed mutagenesis, evaluating the contributions of these residues to thrombin specificity toward protein substrates. Although not identified as naturally occurring variants, differences in these residues are undesirable in a reference material but are unlikely to be present in thrombin produced from prothrombin isolated from blood of individuals with no bleeding disorders. These structural features, *e.g*., exosites and functionally important residues, are shown in Figs. 6–8.

### 2.5 Thrombin Na^+^ Binding Site—Specific Ion–Directed Substrate Specificity

Thrombin possesses a binding site for a single sodium ion that alters thrombin specificity for its substrates [48, 49]. When the Na^+^ site is fully occupied, thrombin specificity and catalytic efficiency are preferentially directed to fibrinogen as the substrate. When unoccupied, the specificity is directed to protein C, *i.e*., the proteinase that functions in stopping the conversion of prothrombin to thrombin by proteolytically inactivating a component (factor Va) of the prothrombin activation complex (prothrombinase) [50, 51]. Figure 7 shows the residues identified as forming the Na^+^ site [52, 53]. While unlikely to be an influence quantity in a reference measurement procedure, mutations in this site are undesirable for a reference material.

### 2.6 Importance of Mutations and Polymorphisms: Effects on Thrombin Biological Activity

Primary structure variants can be classified as “silent or inconsequential,” *i.e*., without detectable effect on the biological activity in the reference measurement procedure, or “consequential,” *i.e.*, with demonstrable effect on the biological activity, *i.e.*, on substrate recognition and proteolytic cleavage efficiency.

Because thrombin is widely recognized as the protease responsible for the conversion of fibrinogen, the soluble protein that circulates in the blood plasma, into the gelatinous fibrin blood clot, this makes fibrinogen an attractive choice for the substrate for a reference measurement procedure. However, fibrinogen is only one of thrombin’s substrates of physiological and medical importance in blood clotting. Consideration of other substrates is necessary prior to making the selection. Thrombin participates in more than six different proteolytic reactions in blood coagulation prior to clot formation [56, 57], and it interacts with cell receptors that do not require proteolysis for response [46, 58, 59].

An important discovery related to thrombin involvement in multiple reactions is that its specificity with respect to its action on protein substrates and cell receptors involves interaction between thrombin and several other plasma proteins. Moreover, only some of these interactions are classical enzyme-substrate interactions; other interactions involve the proteins as effector (regulatory) molecules that alter the catalytic specificity and efficiency of thrombin. From a purely biological knowledge perspective, the multiplicity of interactions of other proteins with thrombin represents a fascinating set of regulatory processes to investigate and to understand. However, from the perspective of development of a reference system for measuring thrombin activity and substance amount, such interacting molecules fall more obviously into the category of influence quantities. Consequently, effects of these proteins on the measurement of thrombin activity should be minimized, and the knowledge regarding them should be used to guide the development of the measurement methods and materials. Some of these influence quantities probably cannot be eliminated entirely from routine methods, but their effects should be minimized in those procedures.

As will be described below, a reference measurement procedure based on the release of the fibrinopeptides from fibrinogen is proposed. However, it cannot be guaranteed that “silent” residues in that reaction will necessarily be silent with respect other protein substrates. In such situations, decreased catalytic efficiency and/or binding affinity (loss of function) and/or increased efficiency (gain of function) could occur and be different depending on the substrate. Rather than being a limitation, the availability of a suitable reference material and measurement procedure can be expected to aid in identifying interactions that otherwise would be missed or discounted because of inadequate reference materials against which to unambiguously make comparison of the new effects. Anticipating what will be discussed later, fibrinogen is the substrate for which interactions with thrombin’s active and extended active site and exosites are best described and understood.

### 2.7 Biological Process in Which Thrombin Is Produced

To permit understanding of the two most common and most problematic structural heterogeneities in thrombin preparations, it is useful to briefly review the process(es) by which thrombin is formed. Thrombin is formed from its circulating precursor, prothrombin, as the consequence of two necessary proteolytic cleavages. Depending upon the protease, or enzyme complex, employed in the conversion of prothrombin to thrombin, intermediates can be formed, with potentially complicating consequences. The relevant product for action on fibrinogen is α-thrombin.

**Fig. 9:**
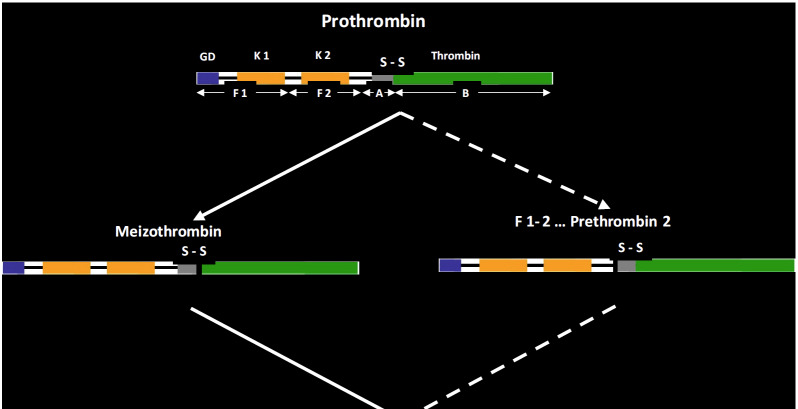
The parallel pathways by which thrombin is produced from prothrombin. The form of thrombin that is active on the multiplicity of its protein substrates is α-thrombin, the final product of this pathway. Depending on the activator (prothrombinase or the snake venom protease used), the intermediate form meizothrombin is formed very transiently (prothrombinase; factor Xa is the active protease), or it can be a primary product (proteases from *Echis carinatus* venom or *Oxyuranus scutellatus* venom). In addition, α-thrombin can be further cleaved to form β- and γ-thrombins—forms that have lost the specificity of α-thrombin and that, when present in α-thrombin preparations, confound the interpretations of thrombin action and severely limit the quality and thus utility of reference materials for thrombin (see Table 1). Figure was modified from Ref. [77].

The pathways by which thrombin is formed from human prothrombin are shown in Fig. 9 [77]. Although the final product is α-thrombin, an intermediate form of thrombin, meizothrombin, is also formed. As the result of factor Xa catalyzed catalysis, meizothrombin is transformed into α-thrombin. Only α-thrombin is an efficient protease for converting fibrinogen into fibrin; meizothrombin is only 1% as effective as α-thrombin [60]. Some snake venom enzymes are also used for preparing thrombin from prothrombin [61, 62]; these produce principally meizothrombin, which then converts to α-thrombin. For producing recombinant thrombin, the thrombin precursor prethrombin 2 is prepared and converted to thrombin by the enzyme from *Echis carinatus* [9–11]. The snake venom enzymes are particularly convenient and more readily available then the complex components of the physiological prothrombin activator (prothrombinase^2^2 Prothrombinase is the name given to the mixture of factor Xa, factor Va, phospholipid vesicles, and Ca^2+^. It is used rarely because few laboratories produce the protein components required for this activator. Proteolysis of prothrombin to form thrombin and the activation fragments can be achieved by factor Xa alone, albeit at such a slow rate that high concentrations of factor Xa are required [77, 104, 1].) and are thus practical and widely used “tools” for converting prothrombin to thrombin [63, 64], particularly in identifying functional defects in patients with coagulation deficiencies.

**Fig. 10:**
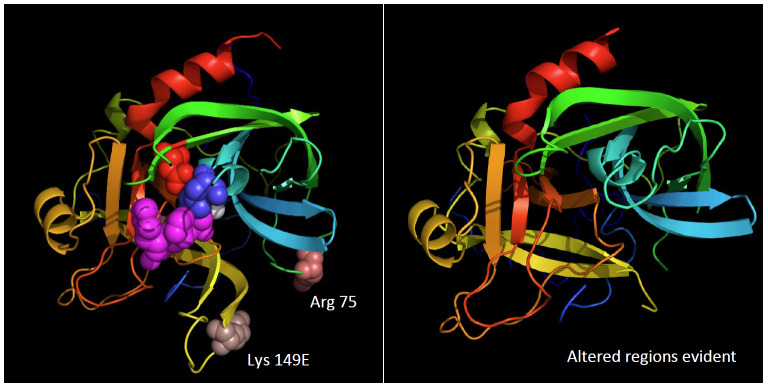
Cleavage sites that convert α-thrombin to β-thrombin and γ-thrombin. Cleavage of α-thrombin at Arg (75, chymotrypsin numbering) produces β-thrombin. Cleavage of β-thrombin at Lys (149E, chymotrypsin numbering) produces γ-thrombin. Data are from Ref. [109]; thrombin structure is 1PPB from Refs. [22, 67].

The most important feature for producing a suitable reference material for α-thrombin is the absence of the proteolytic degradation products: β-thrombin and γ-thrombin. These proteolytically degraded thrombins are less than 0.05% as effective as α-thrombin [65, 66] in cleaving the fibrinopeptides from fibrinogen and inactivation by antithrombin [108]. Structurally, these two degraded forms of thrombin differ in molecular weight from α-thrombin by only one or two molecules of water, respectively (Fig. 10). Preventing the formation of these degraded forms of thrombin and/or eliminating them from preparations of α-thrombin are the most important steps in preparing a suitable reference material for thrombin.

### 2.8 Critical Functional Consequences of Structural Changes in β- and γ-Thrombins

Thrombin’s peptide bond specificity, like that of trypsin, is for the amide bond of basic amino acid residues, primarily arginine and secondarily lysine. All forms of thrombin (meizothrombin, α-thrombin, β-thrombin, and γ-thrombin) hydrolyze arginyl and lysyl peptide bonds in low-molecular-weight synthetic substrates. Because of their convenience, these substrates are attractive for use in assessing thrombin proteolytic activity. However, they do not adequately distinguish between α- and β- or γ-thrombins [68, 69, 106, 107], thus making them of no use for a reference measurement procedure. This is in marked contrast to the different forms of thrombin efficiency in proteolysis of protein substrates. Examples of the large magnitude of the kinetic differences are given in Table 1. However, there are routine methods that involve measurement of generated thrombin using peptide chromogenic or fluorogenic substrates for which they are completely appropriate; these routine methods are not discussed here.

### 2.9 Thrombin “On Paper” Versus Thrombin the Isolated Substance

Some historical context again may be useful for appreciating the technical challenges and requirements for a thrombin reference material if it is to be deemed suitable for its intended use as a highest-order reference material for both substance amount and biological activity.^3^3 This point is reiterated because thrombin preparations of ambiguous or dubious suitability are still employed as reference materials in some methods used in pharmaceutical product evaluation. Isolation of thrombin from blood plasma can be achieved by a variety of procedures. Initially, the most homogeneous preparations have begun with a fraction of mixed plasma proteins, Cohn fraction II + III from ethanol precipitation [70, 71], or a similar fraction prepared by diethyl either precipitation [72]. These were by-products of therapeutic plasma fraction production.

**Table 1:** Comparison of action of α-thrombin and (β,γ)-thrombins on substrates and reaction with inhibitors.

	Catalytic Efficiency, k_c_/K_m_ (L/mol s^−1^)⁴							
**Substrate**	α-Thrombin	β-,-Thrombin	α/(β,γ)	Note			Ref.
Tosyl-Gly-Pro- Arg-pNA	2.6 × 10^7^	1.1 × 10^7^	2.4				[106]
D-Phe-Pip-Arg-pNA	5.5 × 10^7^	4.2 × 10^7^	1.3	γ-thrombin			[105]
Fibrinogen	1.17 × 10^7^	5 × 10^3^	2400	Fibrinopeptide A (FpA) release			[107]
Fibrinogen			>80				[105]
Factor XIII	1.5 × 10^5^	2.6 × 10^4^	5.8	Peptide release			[107]
Antithrombin	1.1 × 10^4^	3.7 × 10^3^	3	Thrombin activity loss			[108]
Antithrombin	1.1 × 10^4^	7 × 10^3^	1.6	Thrombin activity loss			[73]
Antithrombin			≈ 1	Probe displacement			[107]
Protein C			≈ 40	In the presence of co factor thrombomodulin			[113]
**Inhibitor (Exosite 1)**							
Hirudin (β-thrombin)	1.1 × 10^9^	1.7 × 10^7^	6.5	Rapid kinetics			[74]
Hirudin (γ-thrombin)	1.1 × 10^9^	1.3 × 10^4^	85,000				[75]

⁴For hirudin, the constant is the on-rate constant. Reaction conditions and compositions are omitted because the two forms of thrombin were measured under the same conditions.

For biochemical research, precipitates were produced by adding BaCl_2_ to Na citrate anticoagulated plasma or BaSO_4_ to Na oxalate anticoagulated plasma [76, 77]. Such preparations were frequently called “prothrombin” because thrombin would be produced from them [78]. However, these initial products were mixtures of all the vitamin K–related coagulation factors: prothrombin, factors VII, IX, and X, and subsequently identified proteins given the names protein C, protein S, and protein Z [79, 80]. The term “prothrombin complex” has been used to describe these preparations [81, 82]. After “activation,” the capability to cause clotting led to the product being called thrombin, but confusion often resulted from variable results of the use of such “thrombin” forms. After the introduction of ion-exchange cellulose for protein purification, other clotting factors were isolated from the commercial “thrombin” [3, 4]. In consequence, early literature is confusing, and the conclusions drawn from many experiments reported as late as the 1970s are no longer meaningful [83, 124].

Purification to relatively high degrees of homogeneity became possible with the introduction of dextran-based ion exchange, gel filtration media, and the analytical electrophoresis methods such as “disc gel” electrophoresis [84] in the both the absence [137] and presence of sodium dodecyl sulfate [85] in the 1960s and 1970s. The first large-scale purification of vitamin K–dependent coagulation factors that focused on purity (homogeneity) by these procedures were done by two authors of this document [86, 121]. Ignorance of the previously noted insight “…purity is a concept that has no meaning except with reference to the methods and assumptions used in studying the substance…” [12] regrettably resulted in much of the work done on thrombin being of indeterminable validity, and thus that work is now lost in oblivion.

Purification methods, beginning with a Cohn fraction [116] and now chromatography, can produce thrombin of consistent high quality as assessed both by both homogeneity and biological activity (specific activity, *i.e.*, activity units/mass) criteria when calibrated against an international “standard” [6, 87, 88]. However, the analytical data attesting to the quality of the preparations, particularly as they relate to substance amount, are commonly not available, and thus material certified to metrological standards is still not available. Some of the available materials may be entirely suitable, but regrettably the evidence that they are suitable is not available, thus precluding their use in a metrologically rigorous calibration process.

As noted above, methods for preparing thrombin (human) that is homogeneous by the criteria of exclusion of known other proteins have been employed in research laboratories since the 1970s, all following similar purification strategies. Initial preparation of prothrombin that is freed from the other vitamin K–dependent clotting proteins by anion- and cation-exchange chromatography is the common approach [89, 122]. Additionally, “affinity” chromatography using heparin or dextran sulfate linked to commercially available agarose beads has been used both for prothrombin and other vitamin K–dependent clotting factor purification [90] and for thrombin purification after it has been produced by prothrombin activation [105]. Conversion of prothrombin to thrombin is achieved using snake venom proteases as well as the physiological activator prothrombinase. Of the snake venom activators used, those from *Echis carinatus* [100] or *Oxyuranus scutellatus* [98, 99] are most common. A key to avoiding the formation of β- and γ-thrombins is rapid activation and purification to remove the activation fragments that arise from the amino terminal half of prothrombin. Degradation of α-thrombin is believed to be autocatalytic [113], which occurs inefficiently by α-thrombin but relatively rapidly by β- or γ-thrombin once they are detected in the thrombin preparation. Separation of the activation fragments of prothrombin from thrombin is readily achieved by anion-exchange chromatography [122]. Separation of the activation fragments is important because they bind noncovalently to thrombin and alter the catalytic activity of α-thrombin [79].

### 2.10 Purity, Homogeneity, and Heterogeneity of Thrombin Preparations

The combination of anion- and cation-exchange chromatography also offers evidence for the absence of contaminating proteins and prothrombin activation fragments. The two activation fragments are highly anionic and thus bind to the anion-exchange column stationary phase and require high salt concentrations for elution. Thrombin, being cationic, does not bind to an anion exchanger and elutes in the excluded volume of the anion-exchange column at low salt concentration. The prothrombin activation fragments elute in the excluded volume of the cation exchanger, whereas thrombin is eluted with a gradient of salt concentration. Additionally, measurement of biological activity across the peak of eluting protein permits exclusion of protein that does not exhibit constancy of the ratio of activity to mass, with mass concentration commonly estimated by ultraviolet (UV) absorption spectrophotometry. Constancy of this ratio provides another type of evidence for homogeneity of the thrombin.

Reversible, low-molecular-weight inhibitors of thrombin have been used to minimize proteolytic degradation of thrombin during chromatography; however, the most common inhibitor, benzamidine HCl, absorbs UV strongly and thus reduces the precision of protein concentration measurement by UV absorption. Selection of the pH at which thrombin is minimally active for elution also reduces proteolytic degradation. These considerations are noted because it was observed that prothrombin is more susceptible to proteolysis by thrombin during chromatography. Presumably, this is due to conformational alterations associated with binding to the ion-exchange material that result in increased cleavable peptide bond exposure. Based on the combined anion/cation chromatographic separation approach, which in principle “pulls away” contaminants of opposite charge properties, unwanted substances are “pulled away” from the thrombin. This is because during chromatography, the desired protein, if it elutes later from the column than the contaminants, can displace traces of the earlier-eluting unwanted proteins, thus contaminating the desired protein.

**Fig. 11:**
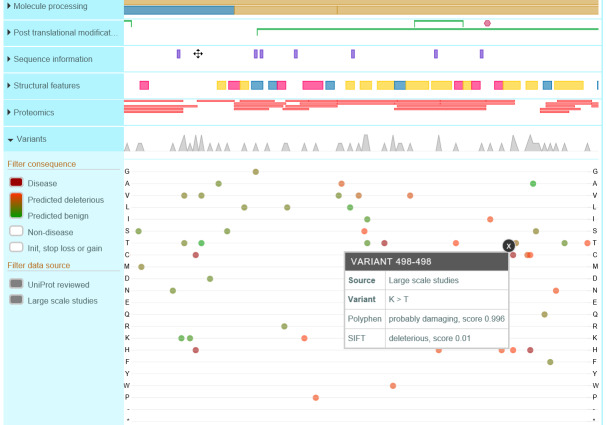
UniProtKB—Documented amino acid variants in thrombin, showing one option in the Feature View display in UniProtKB that identifies variants and suggests a functional consequence of the amino acid substitution.

**Fig. 12:**
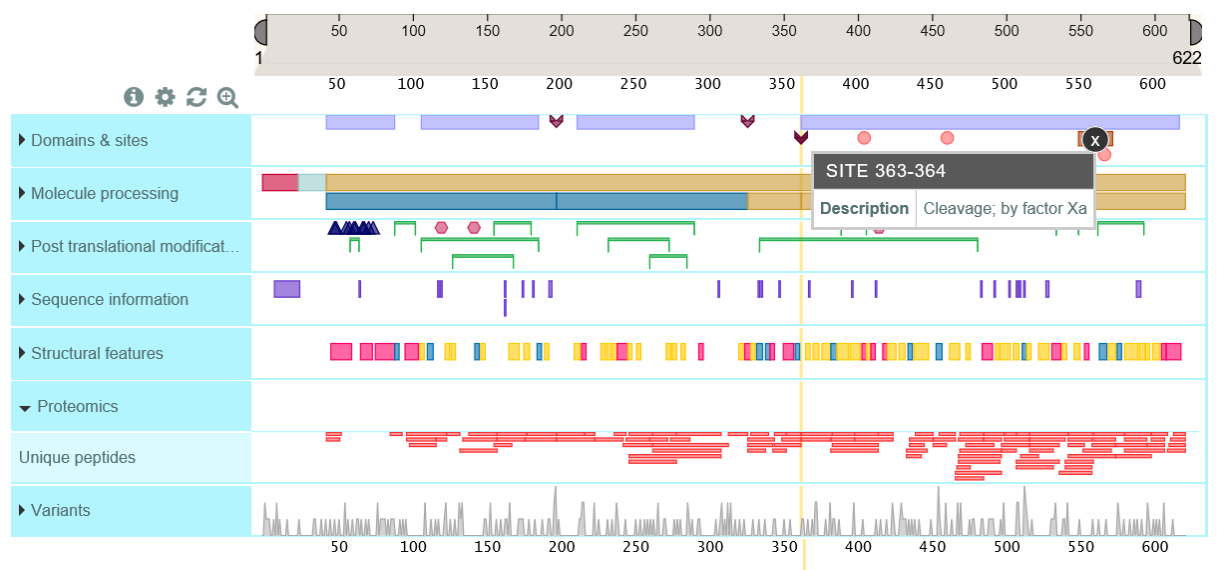
UniProtKB—Diagrammatic presentation of unique peptide after proteolytic digestion. Utility is for predicting MS peptide data that, when combined with the variant data (Fig. 11), can permit targeted examination of a proposed reference material and the possibility of identifying functional consequences from variants present in the material.

Analytical approaches unrelated to chromatographic elution behavior add further evidence for purity, *i.e.*, absence of heterogeneity as related to contamination of other known substances. Electrophoretic separation methods provide evidence for the absence of contaminants, although overloading with respect to α-thrombin is required to achieve detectability of contaminants with these methods. Labeling thrombin at its active histidine site provides a method that increases the sensitivity for detection of α-thrombin degradation because the proteolytic cleavages that produce β- and γ-thrombins occur in the chain containing the active site (the active site containing the heavy chain is also called the B-chain). The β- and γ-thrombins cannot be distinguished, but as the objective is to identify the presence and amount, not the identity, of these degraded forms, this is a powerful method. Derivatized peptide chloromethyl ketones that react with the thrombin active site histidine (Fig. 5) react rapidly with thrombins, are covalently attached, and can have fluorophores and/or radioactive tags attached to facilitate detection and quantification [91]. Thrombin labeled with one such peptide chloromethyl ketone, D-Phe-Pro-Arg-CH_2_Cl, enabled the first crystallization of thrombin for three-dimensional structure determination (Fig. 5). These active site–labeled thrombins are also suitable for peptide mapping by high-performance liquid chromatography (HPLC) with MS detection. Locations of known mutations are illustrated in the following figure. (Fig. 11). Unique peptides from thrombin after controlled proteolysis by trypsin are given in the UniProt database, under Feature View, Proteomics, and they are reproduced in Fig. 12.

### 2.11 Structural Heterogeneity—Polymorphisms and Mutations

Polymorphisms of genetic origin exist that may confound interpretation of the biological function measurements if present in a highest-order reference material. Many have been identified and are noted in the UniProt database, under Feature View, Variants. Mutations identified from functional impairment in individuals with mutations are given, as well as sequence conflicts reported by different laboratories. Based on structural information and inferences, predictions of sequence variant effects on function are given in this section (see also Fig. 11). Most of the variants are predicted to be of minor importance for function; however, only actual data that are traceable to a suitable reference material and method are needed to establish significance or insignificance. Experimental verification would require activity measurement on thrombin isolated from plasma of the individual with the variant or a recombinant product created to possess the variant residue. Such studies are beyond the purview of the development of a traceable reference material for thrombin.

**Fig. 13:**
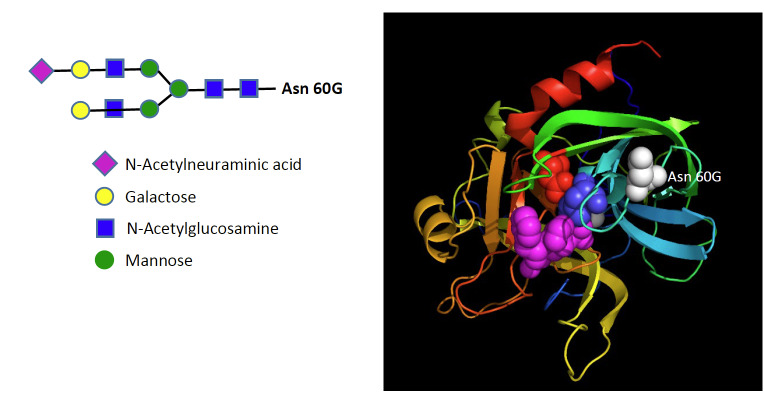
The structure of the oligosaccharide chain at Asn 60G in thrombin, which is the single oligosaccharide chain present in thrombin. No functional consequence has been identified in relation to the oligosaccharide chain. Nomenclature is Asn 60G (chymotrypsin numbering) or Asn 416 (prothrombin numbering). Figure is derived from UniCarbKB, P00734.

An additional source of heterogeneity in thrombin is in the oligosaccharide chain attached to Asn in the thrombin heavy chain (see Figs. 5 and 13). Structurally, the oligosaccharide chain is similar in both human and bovine thrombins; fucosyl residues in the human chain are the major difference [92, 93]. No difference was observed in clotting activity after desialylation and other monosaccharide residue removal, indicating no significance of the oligosaccharide chain in function by this criterion [94, 95]. Charge heterogeneity was observed by isoelectric focusing because of sialic acid residue differences among thrombin molecules [96]; however, no effect on thrombin’s ability to cause clotting was observed. Currently, nothing is known about the consequences of oligosaccharide chain heterogeneity on the immunoassay of thrombin, although immunoassay for thrombin has been employed in research investigations [97].

### 2.12 Physical Characteristics of Human Thrombin

A summary of the considerations addressed above in identifying the substance, and the relevant physical properties useful in the preparation and characterization of the reference material are given in Table 2. Primary structure determinations (amino acid sequence), particularly as reflected in the UniProt database, and three-dimensional structures determined by crystallographers with historical ties to earlier protease crystal structures have resulted in several numbering systems in publications related to thrombin. These alternative numbering systems are detailed in the Appendix.

**Table 2:** Kinetic parameters for α-thrombin action on fibrinogen.

Specificity Constant, k_c_/K_m_ (L/mol s^−1^)	Michaelis Constant, K_m_^c^ (L/mol)	Kinetic Constant, k_c_ (s^−1^)	Reaction Conditions: pH, NaCl (mol/L), Temp (°C), species	Ref.
1.09 × 10^7^ FpA	(7.2 ± 0.9) × 10^−6^	84 ± 04	7.4, 0.137, 37, human	[98, 99]
6.5 × 10^5^ FpB (GPRP)^a^	(7.2 ± 1.5) × 10^−6^	48 ± 5	7.4, 0.137, 37, human	[100]
4.2 × 10^6^ FpB (F_m_)^b^	—	—	7.4, 0.137, 37, human	[154]
Indeterminant, based on clotting time	(13.3 ± 1.1) × 10^−6^	Not measurable	7.5, 0.25, 37, human	[101]
FpA	(11 ± 3) × 10^−6^	79^d^	7.26, 0.3, 25, bovine	[102]
FpB	(6.0 ± 8.5) × 10^−6^	44^d^	7.26, 0.3, 25, bovine	[156]

a The peptide Gly-Pro-Arg-Pro (GPRP) inhibits fibrin monomer (Fm) polymerization.

b Increase in the rate of FpB cleavage by polymerization of fibrin monomers; k_c_/K_m_ increased for FpB release by 6.5 times.

c Values for K_m_ are measured by competition with the chromogenic substrate D-Phe-Pip-Arg-pNA; k_c_ values are not determinable by this method.

d Estimated using 7.9 × 10^−12^ mol/L for the concentration of thrombin given as 1 unit/L.

### 3 Summary

The availability of well-documented methods for purification of the thrombin precursor, prothrombin, and the conversion of prothrombin to thrombin suggests that preparation of the primary, highest-order thrombin reference material and secondary reference materials of essentially equal quality is not only feasible but straightforward enough for widespread use.

Characterization of the reference material is crucial for its utility and widespread acceptance. MS detection after tryptic digestion and peptide separation by HPLC, as an example, will serve to establish definitive data for the certificate of analysis for the reference thrombin. Through isotope- or chromophore/fluorophore-labeled samples, the presence and amount of the two degraded forms of thrombin can be quantified, thus enabling compensation for any interference caused by the presence of these species that may confound a substrate-dependent thrombin activity assay.

### 3.1 Reference Material for Identity

Despite the complexity and the inherent heterogeneities, a metrologically traceable reference material for thrombin is both feasible and can be suitable for its intended use. The extensive data set for thrombin that is the result of decades of structure-function studies provides a satisfactory basis upon which to select an amino acid sequence that identifies and thus defines thrombin as a protein substance. Characterization of the sequence heterogeneities, now entirely practical by MS, enables new observations of structural differences that have functional consequences. This can be done without adversely affecting the utility of the reference material and can enhance the utility of the certificate of analysis for the reference material.

A consensus sequence that broadly reflects the most prevalent amino acid sequence throughout the world is the most desirable. The identification of the differences among populations can assure appropriate compensation for the differences, if required. Through use of the available information on mutations, both naturally occurring and site-directed variants of thrombin can be excluded, if function data indicate that the mutation will cause bias in measurements of thrombin biological activity(ies). Errors in judgment with respect to either loss or gain of function for thrombin action on any of its many substrates can be explicitly described, and, as for any situation of bias (systematic error), a correction can be made for this bias.

### 3.2 Reference Measurement Procedure for Thrombin Proteolytic Activity

Although increasingly observed for many biological macromolecules, the multiplicity of substrates for enzymes creates a challenge in the design of a reference measurement procedure. For thrombin, low-molecular-weight peptides with convenient chromophores for monitoring thrombin-catalyzed hydrolysis as a measure of thrombin activity are appealing. However, substance amount cannot be determined in this manner. Physiologically, thrombin’s target substrates are large protein substrates, *e.g.*, 20 times the mass of thrombin with multiple peptide bonds being cleaved. A key to achieving a high degree of comparability of the measurement results and the inferences made from them is the reference material. The chemical properties described for the thrombin reference material enable inference from activity measurement to substance amount, which must underpin the proposed reference measurement procedure.

### 3.3 Selection of the Most Appropriate Substrate for Measurement of Thrombin Biological Activity

Selection of a single substrate, fibrinogen, that enables definition of the measurand most suitable for assessing thrombin proteolytic activity is most practically justified by eliminating first substrates that are less suitable. The principal criterion used in this proposal is: Proteolytic action on the substrate should reflect as completely as practical contributions of all structural features of the thrombin molecule known to influence the rate of proteolysis. Integrity of the following should all contribute to the activity measurement:

(1) the active site (Fig. 2),
(2) the extended active site (Fig. 8),
(3) the exosites (Fig. 6),
(4) the sodium ion binding site (Fig. 7), and
(5) the presence of the degraded forms of thrombin.

Fibrinogen meets the specified criteria; the reasons why other substrates are unsuitable are detailed below.

Peptide chromogenic or fluorogenic substrates, although they are the most convenient and there exists the largest body of data for thrombin action on them, are disqualified because they measure only the integrity of the active site. They are further disqualified because of their inability to adequately distinguish between α-thrombin and the degraded β- and γ-thrombins. Although usable when the thrombin preparation is the proposed reference material, or when thrombin is indistinguishable from it as indicated by the characterization described for the reference material, the potential for bias and its detection make these substrates unsuitable for a reference measurement procedure.

Of the many protein substrates and receptors upon which thrombin proteolytic action or binding is established to be physiologically relevant, most are substances in low concentration in blood plasma or are located on cell surfaces. Proteins other than fibrinogen are impractical to isolate in the quantities required and are thus unsuitable. Consequently, factor XIII, factor XI, factor V, and factor VIII are eliminated from consideration. Protein C is not excluded; although not a high-concentration plasma protein, it is a by-product of prothrombin isolation and thus could be available. However, its physiologically relevant activation requires the participation of thrombomodulin, a membrane protein only present in small amounts and not readily isolatable in large quantity. Recombinant thrombomodulin could be considered, as could other recombinant proteins. For example, factor VIII could be considered, because it is commercially produced for treatment of hemophilia A. Contemporary preparations of factor VIII, however, are not structurally the same as natural factor VIII because of their greater efficacy, safety, and reduced immunogenicity. Moreover, measurement of the action of thrombin on these other proteins is more complex than for fibrinogen.

### 3.4 Selecting the Reaction That Defines the Measurand

**Fig. 14:**
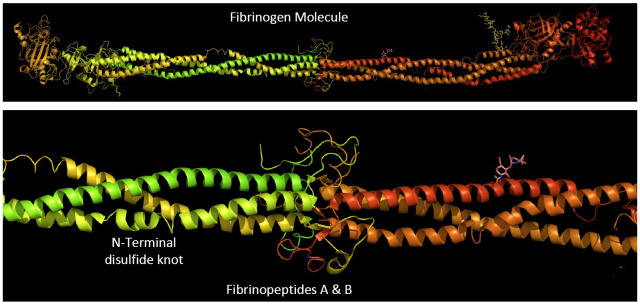
Structure of fibrinogen and fibrinopeptides A and B. Fibrinopeptides A and B comprise a small part of the fibrinogen molecule, *i.e.*, less than 2% of the total polypeptide sequence. Removal of the peptides exposes the polymerization site that interacts with the “knobs” at the ends of the structure to form fibrils. Derived from Refs. [105, 106].

**Fig. 15:**
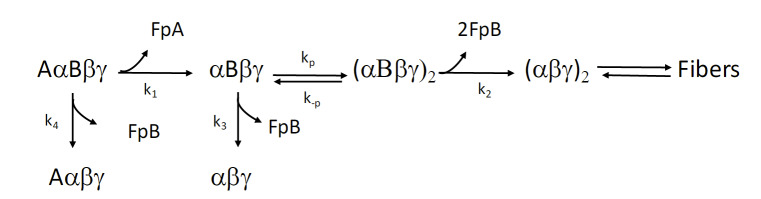
Reaction pathway for release of fibrinopeptides. Fibrinopeptide A (16 residues) is cleaved prior to fibrinopeptide B (14 residues), implying that exposure of fibrinopeptide B must precede its cleavage from the B-chain of fibrin monomer, the product of fibrinopeptide A release. Derived from Ref. [107].

The criteria for selection of the substrate for a reference measurement procedure are best met by fibrinogen. The proteolytic cleavage of fibrinopeptides A and B produces two low-molecular-weight products that are readily separated from the other product, fibrin monomer, and several HPLC methods for quantifying the fibrinopeptides have been reported [154, 103, 104]. Because the peptides are 16 (fibrinopeptide A) and 14 (fibrinopeptide B) amino acid residues in length, internal standards for HPLC and MS can be prepared by chemical synthesis for use and validation of the HPLC methods. Figure 14 displays the crystallographic-determined structure of fibrinogen. Figure 15 details the reaction of thrombin on fibrinogen, revealing the small changes that enable polymerization of fibrin monomer.

### 3.5 Established Kinetic Behavior of the Thrombin-Catalyzed Release of Fibrinopeptides

Consistent behavior for thrombin-catalyzed fibrinopeptide release has been reported over several decades, although the instrumentation and identification methods have been greatly refined over that period. The procedure outlined below is taken from Ref. [108] with only minor adaptations.

Values of the Michaelis constant and the maximum rate of reaction for both human and bovine proteins are listed in Table 2. Concentrations of thrombin and fibrinogen for the measurement are selected to simplify the kinetics of the reaction and thus make analysis of the concentration of the fibrinopeptide(s) versus time simple [153, 165]. Further, when the concentration of the substrate is <0.2 K_m_ for thrombin, and the concentration of thrombin is much less than the fibrinogen concentration, the kinetic behavior is pseudo–first order. Although the action of thrombin on fibrinogen and the process of its transformation from soluble, circulating protein to the gelatinous clot have been long studied, the technological developments and elucidation of the kinetic mechanism in the 1970s through 1990s enabled this straightforward, rigorous procedure to be produced (Fig. 15).

Reaction composition, reactant concentrations, and solution composition employed are summarized in Table 3. The identification of the Na^+^ binding site on thrombin [87, 155, 109] suggests that the NaCl concentration should be increased to 0.2 mol/L to ensure that thrombin is in the “fast form.” Inclusion of the tetrapeptide Gly-Pro-Arg-Pro [110, 111] inhibits polymerization and reduces any complicating effects of fibrin polymerization. However, release of fibrinopeptide B is affected [152, 153, 112], although, if fibrinopeptide A is exclusively measured, this will be of no significance.

**Table 3:** Reactant and reagent compositions for α-thrombin action on fibrinogen.

Reactant or Component	Concentration (in Reaction)	Notes	Ref.
Fibrinogen	2 μmol/L	< 0.2 × K_m_, reaction is dependent on [fibrinogen]	[164]
α-thrombin	1 nmol/L	[thrombin] << [fibrinogen], pseudo–first-order kinetic behavior	[163]
NaCl	0.2 mol/L	[Na^+^] to ensure all thrombin is in the “fast form”	[83, 165]
Tris/HEPES	0.05 mol/L	Increasing buffer capacity for both acidic/alkaline shifts	[113]
H^+^	pH 7.8	Slight plateau in pH dependence curve; closer to plasma pH than maximum/plateau at pH ≈ 8	[114]
PEG 8000	0.1% (mass fraction)	Competing adsorbate to prevent loss of thrombin by adsorption to reaction vessel surface	[169,170, 115]
Fibrinopeptide A (deuterium label)		Internal standard for HPLC quantification	[116]

Reactions are at 37.0 °C, the conventional temperature for enzyme assay. Reaction vessels are preferably polypropylene that has been coated with PEG 20,000 [170, 171]. Polypropylene microcentrifuge tubes (1.8 mL) are convenient for quenching and centrifugally removing precipitated fibrinogen and fibrin monomer. The first solution to be placed in the reaction vessel is fibrinogen to further minimize thrombin adsorption loss. The thrombin in solution is most stable at pH 6.5 in 0.5 mol/L NaCl [116, 117]. The reaction is quenched with 3 mol/L perchloric acid. The buffers listed above are Tris (2-amino-2-hydroxymethyl-propane-1,3-diol) and HEPES (4-(2-hydroxyethyl)-1-piperazineethanesulfonic acid).

Linearity with thrombin concentration, which is expected for simple enzyme-catalyzed reactions, is observed under the conditions of this method [163, 164]. Linearity with fibrinogen concentration is achieved by the selection of the concentration to be <0.2 K_m_ [164]. The greatest advantage of this simplified kinetic behavior is that any laboratory with the HPLC equipment could perform the reference measurement procedure; reliance on special reference measurement service laboratories [117] would then not be required.

The release of both fibrinopeptides A and B under the conditions of the method as published here is described by the rate equations provided in Refs. [152, 53]. The kinetic equations for the full-time course of the reaction are also given in the published reports on which the procedure described above is based [152, 164]. Contemporary personal computer software now exists that can be used to fit the entire reaction time course, thus making it possible to eliminate the restrictions on reactant concentrations that are required for simple linear fibrinopeptide release with time. However, biasing because of contaminants in the fibrinogen preparations and other interactions can complicate the kinetic behavior observed in full reaction time course monitoring and thus influence the measurement results.

For metrological traceability to the International System of Units (SI), the activity of thrombin must be expressed in the appropriate SI unit, the katal [118]. This is readily done and thus provides metrological traceability to the SI; however, the katal has a unique value only under the specified conditions of the reference measurement procedure.

### 3.6 Fibrinogen Preparations—Suitability for the Reference Measurement Procedure

Fibrinogen present in plasma, at concentrations between 200 and 400 mg/dL [119], is a practical choice because of its convenient availability and the large amounts that are available. Methods for purification of fibrinogen suitable for transfusion are varied; the simplest and best characterized date back to the 1960s. Commonly, fibrinogen “quality” is described by the percentage of the fibrinogen protein preparation that is capable of clotting; the clot is manually removed and weighed [120, 121]. Although fibrinogen is commercially available from multiple sources, freedom of the material from factor XIII and plasminogen is important to avoid the complication of cross-linking (factor XIIIa) or proteolytic degradation by plasmin (plasminogen contamination). Other potential contaminants that could affect the quality of the fibrinogen substrate in the reference procedure are the thrombin-activated fibrinolysis inhibitor (TAFI), and tissue plasminogen activator. Changes due to the action of these enzymes on stored or suboptimally handled fibrinogen preparations can be minimized or avoided by suitably “pure” fibrinogen preparations.

In the time interval over which fibrinopeptide release is being measured, minor contaminants are unlikely to influence the behavior of the reaction. Fibrinogen preparations of different purity have been investigated and found to be suitable for use in measurement of fibrinopeptide release [154].

### 3.7 Reaction Conditions, Influence Quantities, and Avoidance of Measurement Bias

#### 3.7.1 Proton Binding—pH

As recognized for all enzymatic reactions, adequate buffering for kinetic measurements of fibrinopeptide release from fibrinogen is required. Optimum pH, for both fibrinogen and peptide p-nitroanilide substrates, is at pH 8.0; an inflection is observed at pH 7.8 in 0.1 mol/L NaCl (ionic strength, 0.15 mol/L) [169, 12]. Based on the near identity of the pH dependence for both types of substrate, the effect appears to be predominately on thrombin. The results from several laboratories using different methods for monitoring the hydrolytic reaction illustrate the consistency of pH dependence [163, 169, 123, 124]. Rationale for the selection of the most advantageous pH can be: (1) minimization of the effects on the measured reaction rate or (2) a pH at which buffer capacity minimizes changes in pH. The latter was the criterion used in studies of the pH dependence of bovine thrombin on peptide p-nitroanilide substrates [111, 169, 125]. The value of pH is frequently selected to be that of plasma, pH 7.4 [154, 163, 164, 168]. However, this is a region of pH dependence with a high slope, and thus it is potentially subject to pH variability that unnecessarily contributes to measurement uncertainty.

#### 3.7.2 Ionic Strength—Electrolyte Identity and Concentration Dependence

It is necessary to select a Na^+^ concentration that is appropriate for all thrombin being in the “fast form” (the optimal conformer for action on fibrinogen) but that minimizes thrombin action on thrombomodulin-related activation of protein C. Using peptide p-nitroanilide substrates acting on bovine thrombin, K_m_ increases by 1.5 between (0.1 and 0.2) mol/L NaCl at pH 7.8; k_c_ is unchanged. Between (0.2 and 0.5) mol/L NaCl, neither K_m_ nor k_c_ is changed [169]. Because of the specific ion effect of Na^+^ (see below), NaCl concentration needs to be >0.2 mol/L [126] to ensure that all thrombin is in the optimal conformer for fibrinopeptide release. The residues implicated (Fig. 7) as well water molecules in the “site” are thus controlled, and any effects of them are minimized.

#### 3.7.3 Na^+^ Effects on Thrombin Proteolytic Activity—Optimizing Specificity

The two forms of thrombin that are modulated by Na^+^ binding [81, 87] differ in their relative specificity on fibrinogen and other protein substrates (Fig. 7). Selecting the NaCl concentration to exceed 0.2 mol/L forces the thrombin into the “fast form,” the form best suited for measuring thrombin proteolytic activity on fibrinogen and synthetic substrates. Alteration of thrombin activity and stability by other ions has also been reported [82, 127, 128].

#### 3.7.4 Divalent Cations—Ca^2+^ Ions and Thrombin Action on Other Substrates

Two effects of Ca^2+^ on the proteolysis of fibrinogen by thrombin require consideration. First, Ca^2+^ binding to fibrinogen affects fibrinopeptide B release; fibrinopeptide A release is minimally affected [129, 130, 131]. Fibrinogen is stabilized by Ca^2+^ [187, 132, 133], and so it might be important to have Ca^2+^ present for fibrinogen to be used over long periods of time. Second, activation of factor XIII is dependent on Ca^2+^, suggesting that the absence of Ca^2+^ can minimize any bias that might occur if the fibrinogen were contaminated with factor XIII. There is no effect of Ca^2+^ on thrombin kinetic parameters for peptide p*-*nitroanilide substrates when assessed with thrombin (bovine) [169]. In the proposed reference procedure, bias from the presence of Ca^2+^ is unlikely; however, differences in the results from routine methods in which thrombin is used (commonly for turbidimetric measurement of fibrinogen) should be expected between Ca^2+^-containing and Ca^2+^-free solutions.

#### 3.7.5 Protein Substrates Other than Fibrinogen—Alternative Substrates as Competitive Inhibitors

Thrombin is a protease with a substantial number of biologically important substrates. The approach to a reference measurement procedure described here is unlikely to suffer bias because of the presence of small amounts of these protein substrates, first, because of efforts to remove even trace amounts from the reference material, and second, because of the selection of the conditions under which thrombin proteolysis of fibrinogen is measured. However, when assigning values for thrombin substance amount and catalytic activity to calibrators along the traceability chain, *i.e.*, calibrators that will be used in routine methods, these other substrates may be important. Some of the more well-known alternative substrates [134] of thrombin are listed in Table 4.

**Table 4:** Limited list of competing substrates and protein inhibitors of thrombin.

Substrate	Name/Function	Ref.
Fibrinogen	Precursor of clot	[135, 136]
Factor XIII	Plasma transglutaminase; cross-links fibrin in clot	[168]
Protease-activated receptors (PARs) 1, 3, 4	Protease-activated receptors, found on cells, *e.g.*, platelets	[95, 96]
Factor V	Component of prothrombinase; catalyzes factor Xa cleavage of peptide bonds in prothrombin	[26, 77, 191]
Factor VIII	Component of factor X activation complex; catalyzes factor IXa cleavage of peptide bonds in factor X	[26, 191]
Factor XI	Factor XIa activates factor IX; sulfated polysaccharides can catalyze the reaction	[92, 137]
TAFI	Thrombin-activated fibrinolysis inhibitor	[138]
Antithrombin	Serine protease inhibitor of thrombin, factors Xa, IXa, VIIa; inactivation reaction is catalyzed by heparin with unique pentasaccharide sequence	[74]
Heparin cofactor II	Serine protease inhibitor of thrombin; catalyzed by sulfated polysaccharides; no specific saccharide sequence required	[139]

As influence quantities, alternative substrates are potential sources for bias. The extent to which these substrates create bias in a measurement result must be investigated whenever suspected. Although commutability is most frequently discussed as a matrix-related feature, the alternative substrates noted here are present when the matrix is blood plasma. When the alternative substrates interfere to such an extent that they are readily detectable, correction for them can be made by treating their reactions as conventional, parallel enzymatic reactions or as competitive inhibitors. An example of this is thrombin inactivation by its inhibitors, antithrombin, α-1 antitrypsin (α-1 proteinase inhibitor), and α-2 macroglobulin. This situation is noted in the entries in Table 4 and the references cited there.

A recently published routine method addressing interferences has the potential to improve the accuracy and help establish the metrological traceability of clinical measurement results for thrombin activity in plasma [140].

#### 3.7.6 Thrombin Adsorption Loss and Adsorption Prevention

Thrombin action on fibrinogen, synthetic substrates, and other protein substrates, whatever the “signal” for monitoring the reaction, occurs at concentrations of thrombin at nanomoles per liter or lower [107, 152, 164]. Under these conditions, thrombin is rapidly adsorbed, and, depending on the surface, it can be irreversibly adsorbed. Adsorbed thrombin becomes inactive, thus changing its concentration in the reaction. Buffer solutions have commonly included plasma albumin of variable purity as a competing adsorbate; although effective in reducing adsorption loss, albumin prepared by some methods can be contaminated with enzymes and metal ions. Therefore, human albumin of drug quality is preferred.

Polyethylene glycol has been shown to be a better competing adsorbate and may stabilize thrombin as well as prevent adsorption [170]. Whenever possible, polypropylene containers, precoated with high-molecular-weight polyethylene glycol [171], have been found to be the most suitable for reaction vessels and for preparing dilutions of thrombin at higher concentrations than employed in the reactions.

## 4 Discussion—A Traceability Scheme for Thrombin with Both Metrological and Philosophical Uncertainty

A metrologically ideal measurement system includes both a reference material and a reference measurement procedure [141]. Without both, traceability to substance identity and measurement of substance amount and structure-derived biological activity cannot be achieved. In contrast to simpler substances, the inherent heterogeneity in the structure of biological macromolecules makes traceability to a unique chemical substance difficult to achieve, although it is possible as argued here by a consensus definition. In addition to structural heterogeneity, material heterogeneity or “purity,” as expressed by the absence of contaminating substances, presents a challenge of perhaps greater magnitude. The potency of even extremely small amounts of contaminating substances in biological processes places great demand on the sensitivity of the analytical methods used to detect and quantify the contaminants, and, when a contaminant is below the limit of detection, a biological assay that detects an effect of the contaminant will require some further characterization. Because of the impossibility of “proving” the absence (nonexistence) of a trace contaminant, and the existence of multiple substrates, *e.g.*, as noted for the enzyme thrombin, the possibility of discovery of a previously unidentified contaminant must always be entertained and, when evidence suggests, investigated.

Metrological traceability extends beyond the limited portion of the chain illustrated in Fig. 1 [142]. In fact, the utility of the traceability chain lies in obtaining comparability of measurement results from procedures for routine use because of the employment of those results in medical and commercial decision making.

Modifications to the traceability chain may be required, but the modified chain must retain its link to fundamental SI units: kilogram, mole, and katal. The hierarchy of measurement steps necessary for assigning the measurand value to the final material and using the material with methods used in routine laboratory medicine must be unambiguous, with the caveats related to influence quantities in the sample analyzed in a routine method.

### 4.1 Metrological Confidence—Recognition of Inherent Limitations for Biological Measurands

In situations involving macromolecules of biological origin, classical assessment of measurement uncertainty may not be practical. While uncertainties can be quantitatively estimated for the results of measurements of well-defined measurands, the fitness of a complex multifunctional material for a specific purpose may also depend on poorly defined properties and interactions that can only be qualitatively assessed. As an alternative to metrological uncertainty, the concept of metrological confidence can be employed as a means of evaluating the fitness of a material or measurement procedure. This view is concordant with that of the Intergovernmental Panel on Climate Change Working Group III (IPCC WG III): “Where uncertainty is assessed qualitatively, it is characterized by providing a relative sense of the amount and quality of evidence (that is, information from theory, observations or models indicating whether a belief or proposition is true or valid) and the degree of agreement (that is, the level of concurrence in the literature on a particular finding). This approach is used by WG III through a series of self-explanatory terms such as: high agreement, much evidence; high agreement, medium evidence; medium agreement, medium evidence; *etc*.” [143].

**Fig. 16:**
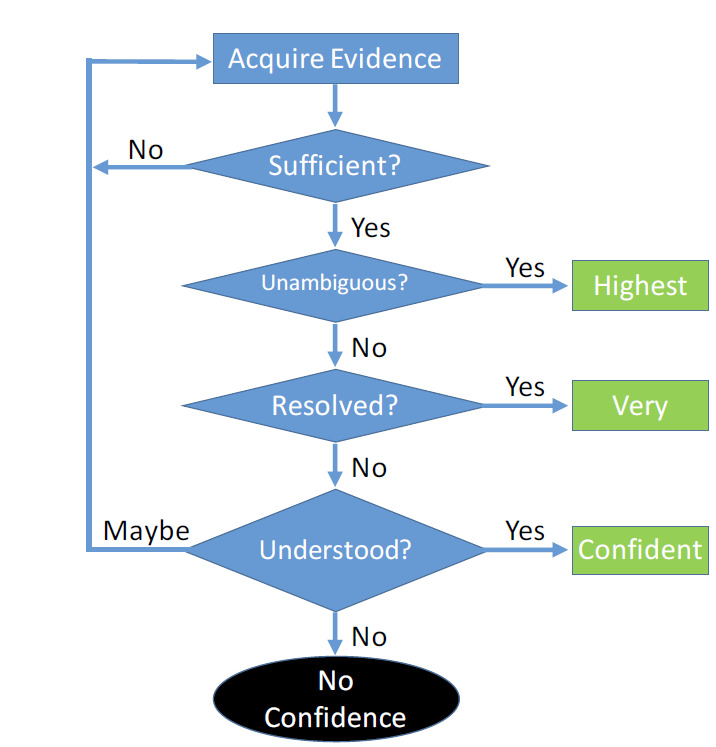
Metrological confidence—a reference system for thrombin. An evaluation process to enable meaningful interpretation of results when metrological uncertainty cannot be quantitatively estimated is suggested for evaluating the proposed reference system for thrombin. Consensus of the stakeholders who are the intended beneficiaries determines the level of confidence that will be assigned to the reference material and the measurement procedure [144].

Metrological confidence (Fig. 16) in the reference system for thrombin can be asserted from the following considerations:

(1) General concordance in the sequence for human α-thrombin from multiple methods and crystallographic structures.
(2) Identification of mutations and polymorphisms that are consequential for thrombin’s biological activity.
(3) Established methods for purification of the precursor to thrombin that provide evidence for the separation of contaminating substances that influence the measurement of thrombin activity.
(4) Availability of technologies capable of detecting and quantifying contaminants, *e.g.*, protein and peptide MS.
(5) Selection of the substrate, fibrinogen, that utilizes several sites and exosites within the thrombin molecule for recognition, specificity, and catalytic efficiency.
(6) Choice of reaction solution composition and component concentrations that minimize measurement bias and imprecision.
(7) Kinetic characterization of the reaction mechanism that enables detailed characterization of the reference material and the substrate beyond the conditions specified for the measurement procedure.

Reference materials have been provided for measurement of thrombin activity for decades, with advances in understanding and methods for assessment of quality employed with each successive preparation [7]. The geometric mean of the results from a variety of measurement methods widely used by laboratories throughout the world represents a consensus value for units assigned to these reference standards. Dose response behavior is taken to be represented by the log of the response in the assay method versus the log of the concentration of the dilution of the reference materials. The most recent material was compared to both the WHO’s 1st International Standard and the NIH’s U.S. Standard, Lot J, to produce a single unit for the WHO 2nd International Standard 01/580. In deciding the best value to be assigned to the material, because of the recognized bias that would result from β- and γ-thrombin activity measured by peptide chromogenic substrates, data from chromogenic substrate hydrolysis were excluded [7].

Preparations of thrombin used in research laboratories have been characterized with respect to the unit activity provided by WHO and NIH reference materials. The ratios of activity to protein mass (commonly measured spectrophotometrically) have had specific activities from (3000 to 4000) units/mg protein. By active site titration, these materials have been generally 95% active (esterolytically active as measured by p*-*nitrophenyl-guanidinobenzoate burst). International Standard 01/580 is indicated to be 2240 units/mg [7]. Methods for protein quantification are not indicated, and thus overestimation of protein, which would underestimate the specific activity, is not possible. If the measured protein is “inert” and would not create bias, this international standard might be all α-thrombin; this is, however, not determinable from the published data.

Based on information provided in this document, we conclude that the development of both a reference material and reference measurement procedure that are suitably characterized and metrologically traceable for substance amount and proteolytic activity of thrombin is entirely feasible. The production of such a product would be beneficial for calibration and value assignment in both laboratory diagnostic and therapeutic products.

The potential utility of a reference system for thrombin is great. Licensed thrombin products for arresting bleeding are already in use. Some of these are potentially available and might be suitable for secondary reference materials to be used in routine laboratory methods that employ thrombin. Although perhaps brash, we suggest that the lessons learned from the development of this material and method could inform the development of other reference systems, and thus improve measurement comparability for other biological materials of clinical importance.

## 5 Appendix

**Table A1:** Thrombin numbering variations for the A (“light”) chain.

UniProt (Includes Signal, Propeptide)	Prothrombin Amino-Terminus, Residue 1	Chymotrypsin System	One-Letter Amino Acid Code	Three-Letter Amino Acid Code	Thrombin Chain Numbering
328	285	1 H	T	Thr	1
329	286	1 G	F	Phe	2
330	287	1 F	G	Gly	3
331	288	1 E	S	Ser	4
332	289	1 D	G	Gly	5
333	290	1 C	E	Glu	6
334	291	1 B	A	Ala	7
335	292	1 A	D	Asp	8
336	293	1	C	Cys	9
337	294	2	G	Gly	10
338	295	3	L	Leu	11
339	296	4	R	Arg	12
340	297	5	P	Pro	13
341	298	6	L	Leu	14
342	299	7	F	Phe	15
343	300	8	E	Glu	16
344	301	9	K	Lys	17
345	302	10	K	Lys	18
346	303	11	S	Ser	19
347	304	12	L	Leu	20
348	305	13	E	Glu	21
349	306	14	D	Asp	22
350	307	14 A	K	Lys	23
351	308	14 B	T	Thr	24
352	309	14 C	E	Glu	25
353	310	14 D	R	Arg	26
354	311	14 E	E	Glu	27
355	312	14 F	L	Leu	28
356	313	14 G	L	Leu	29
357	314	14 H	E	Glu	30
358	315	14 I	S	Ser	31
359	316	14 J	Y	Tyr	32
360	317	14 K	I	Ile	33
361	318	14 L	D	Asp	34
362	319	14 M	G	Gly	35
363	320	15	R	Arg	36

**Table A2:** Thrombin numbering variations for the B (“heavy”) chain.

UniProt (Includes Signal, Propeptide)	Prothrombin Amino-Terminus, Residue 1	Chymotrypsin System	One-Letter Amino Acid Code	Three-Letter Amino Acid Code	Thrombin Chain Numbering
364	321	16	I	Ile	37
365	322	17	V	Val	38
366	323	18	E	Glu	39
367	324	19	G	Gly	40
368	325	20	S	Ser	41
369	326	21	D	Asp	42
370	327	22	A	Ala	43
371	328	23	E	Glu	44
372	329	24	I	Ile	45
373	330	25	G	Gly	46
374	331	26	M	Met	47
375	332	27	S	Ser	48
376	333	28	P	Pro	49
377	334	29	W	Trp	50
378	335	30	Q	Gln	51
379	336	31	V	Val	52
380	337	32	M	Met	53
381	338	33	L	Leu	54
382	339	34	F	Phe	55
383	340	35	R	Arg	56
384	341	36	K	Lys	57
385	342	36 A	S	Ser	58
386	343	37	P	Pro	59
387	344	38	Q	Gln	60
388	345	39	E	Glu	61
389	346	40	L	Leu	62
390	347	41	L	Leu	63
391	348	42	C	Cys	64
392	349	43	G	Gly	65
393	350	44	A	Ala	66
394	351	45	S	Ser	67
395	352	46	L	Leu	68
396	353	47	I	Ile	69
397	354	48	S	Ser	70
398	355	49	D	Asp	71
399	356	50	R	Arg	72
400	357	51	W	Trp	73
401	358	52	V	Val	74
402	359	53	L	Leu	75
403	360	54	T	Thr	76
404	361	55	A	Ala	77
405	362	56	A	Ala	78
406	363	57	H	Hs	79
407	364	58	C	Cys	80
408	365	59	L	Leu	81
409	366	60	L	Leu	82
410	367	60 A	Y	Tyr	83
411	368	60 B	P	Pro	84
412	369	60 C	P	Pro	85
413	370	60 D	W	Trp	86
414	371	60 E	D	Asp	87
415	372	60 F	K	Lys	88
416	373	60 G	N	Asn	89
417	374	60 H	F	Phe	90
418	375	60 I	T	Thr	91
419	376	61	E	Glu	92
420	377	62	N	Asn	93
421	378	63	D	Asp	94
422	379	64	L	Leu	95
423	380	65	L	Leu	96
424	381	66	V	Val	97
425	382	67	R	Arg	98
426	383	68	I	Ile	99
427	384	69	G	Gly	100
428	385	70	K	Lys	101
429	386	71	H	His	102
430	387	72	S	Ser	103
431	388	73	R	Arg	104
432	389	74	T	Thr	105
433	390	75	R	Arg	106
434	391	76	Y	Tyr	107
435	392	77	E	Glu	108
436	393	77 A	R	Arg	109
437	394	78	N	Asn	110
438	395	79	I	Ile	111
439	396	80	E	Glu	112
440	397	81	K	Lys	113
441	398	82	I	Ile	114
442	399	83	S	Ser	115
443	400	84	M	Met	116
444	401	85	L	Leu	117
445	402	86	E	Glu	118
446	403	87	K	Lys	119
447	404	88	I	Ile	120
448	405	89	Y	Tyr	121
449	406	90	I	Ile	122
450	407	91	H	His	123
451	408	92	P	Pro	124
452	409	93	R	Arg	125
453	410	94	Y	Tyr	126
454	411	95	N	Asn	127
455	412	96	W	Trp	128
456	413	97	R	Arg	129
457	414	97 A	E	Glu	130
458	415	98	N	Asn	131
459	416	99	L	Leu	132
460	417	100	D	Asp	133
461	418	101	R	Arg	134
462	419	102	D	Asp	135
463	420	103	I	Ile	136
464	421	104	A	Ala	137
465	422	105	L	Leu	138
466	423	106	M	Met	139
467	424	107	K	Lys	140
468	425	108	L	Leu	141
469	426	109	K	Lys	142
470	427	110	K	Lys	143
471	428	111	P	Pro	144
472	429	112	V	Val	145
473	430	113	A	Ala	146
474	431	114	F	Phe	147
475	432	115	S	Ser	148
476	433	116	D	Asp	149
477	434	117	Y	Tyr	150
478	435	118	I	Ile	151
479	436	119	H	His	152
480	437	120	P	Pro	153
481	438	121	V	Val	154
482	439	122	C	Cys	155
483	440	123	L	Leu	156
484	441	124	P	Pro	157
485	442	125	D	Asp	158
486	443	126	R	Arg	159
487	444	127	E	Glu	160
488	445	128	T	Thr	161
489	446	129	A	Ala	162
490	447	129 A	A	Ala	163
491	448	129 B	S	Ser	164
492	449	129 C	L	Leu	165
493	450	130	L	Leu	166
494	451	131	Q	Gln	167
495	452	132	A	Ala	168
496	453	133	G	Gly	169
497	454	134	Y	Tyr	170
498	455	135	K	Lys	171
499	456	136	G	Gly	172
500	457	137	R	Arg	173
501	458	138	V	Val	174
502	459	139	T	Thr	175
503	460	140	G	Gly	176
504	461	141	W	Trp	177
505	462	142	G	Gly	178
506	463	143	N	Asn	179
507	464	144	L	Leu	180
508	465	145	K	Lys	181
509	466	146	E	Glu	182
510	467	147	T	Thr	183
511	468	148	W	Trp	184
512	469	149	T	Thr	185
513	470	149 A	A	Ala	186
514	471	149 B	N	Asn	187
515	472	149 C	V	Val	188
516	473	149 D	G	Gl	189
517	474	149 E	K	Lys	190
518	475	150	G	Gly	191
519	476	151	Q	Gln	192
520	477	152	P	Pro	193
521	478	153	S	Ser	194
522	479	154	V	Val	195
523	480	155	L	Leu	196
524	481	156	Q	Gln	197
525	482	157	V	Val	198
526	483	158	V	Val	199
527	484	159	N	Asn	200
528	485	160	L	Leu	201
529	486	161	P	Pro	202
530	487	162	I	Ile	203
531	488	163	V	Val	204
532	489	164	E	Glu	205
533	490	165	R	Arg	206
534	491	166	P	Pro	207
535	492	167	V	Val	208
536	493	168	C	Cys	209
537	494	169	K	Lys	210
538	495	170	D	Asp	211
539	496	171	S	Ser	212
540	497	172	T	Thr	213
541	498	173	R	Arg	214
542	499	174	I	Ile	215
543	500	175	R	Arg	216
544	501	176	I	Ile	217
545	502	177	T	Thr	218
546	503	178	D	Asp	219
547	504	179	N	Asn	220
548	505	180	M	Met	221
549	506	181	F	Phe	222
550	507	182	C	Cys	223
551	508	183	A	Ala	224
552	509	184	G	Gly	225
553	510	184 A	Y	Tyr	226
554	511	185	K	Lys	227
555	512	186	P	Pro	228
556	513	186 A	D	Asp	229
557	514	186 B	E	Glu	230
558	515	186 C	G	Gly	231
559	516	186 D	K	Lys	232
560	517	187	R	Arg	233
561	518	188	G	Gly	234
562	519	189	D	Asp	235
563	520	190	A	Ala	236
564	521	191	C	Cys	237
565	522	192	E	Glu	238
566	523	193	G	Gly	239
567	524	194	D	Asp	240
568	525	195	S	Ser	241
569	526	196	G	Gly	242
570	527	197	G	Gly	243
571	528	198	P	Pro	244
572	529	199	F	Phe	245
573	530	200	V	Val	246
574	531	201	M	Met	247
575	532	202	K	Lys	248
576	533	203	S	Ser	249
577	534	204	P	Pro	250
578	535	204 A	F	Phe	251
579	536	204 B	N	Asn	252
580	537	205	N	Asn	253
581	538	206	R	Arg	254
582	539	207	W	Trp	255
583	540	208	Y	Tyr	256
584	541	209	Q	Gln	257
585	542	210	M	Met	258
586	543	211	G	Gly	259
587	544	212	I	Ile	260
588	545	213	V	Val	261
589	546	214	S	Ser	262
590	547	215	W	Trp	263
591	548	216	G	Gly	264
592	549	217	E	Glu	265
593	550	218	G	Gly	266
594	551	219	C	Cys	267
595	552	220	D	Asp	268
596	553	221	R	Arg	269
597	554	222	D	Asp	270
598	555	223	G	Gly	271
599	556	224	K	Lys	272
600	557	225	Y	Tyr	273
601	558	226	G	Gly	274
602	559	227	F	Phe	275
603	560	228	Y	Tyr	276
604	561	229	T	Thr	277
605	562	230	H	His	278
606	563	231	V	Val	279
607	564	232	F	Phe	280
608	565	233	R	Arg	281
609	566	234	L	Leu	282
610	567	235	K	Lys	283
611	568	236	K	Lys	284
612	569	237	W	Trp	285
613	570	238	I	Ile	286
614	571	239	Q	Gln	287
615	572	240	K	Lys	288
616	573	241	V	Val	289
617	574	242	I	Ile	290
618	575	243	D	Asp	291
619	576	244	Q	Gln	292
620	577	245	F	Phe	293
621	578	246	G	Gly	294
622	579	247	E	Glu	295
					

**Glossary**
1PPB Protein Data Bank designation for thrombin (human coordinates from X-ray structure)
1TRN Protein Data Bank designation for trypsin (human)
Arg arginine
Factor II prothrombin, sometimes simply designated “Pro” in coagulation literature
Factor V precursor of the nonenzymatic catalyst factor Va, a component of prothrombinase, the complex activator that converts prothrombin to thrombin. Cleavage of factor V to form factor Va is catalyzed by thrombin and less efficiently by factor Xa
Factor Va see factor V
Factor VIIa protease catalyzing cleavage of factor X to form factor Xa, the protease that cleaves prothrombin. Factor VII is the first protease precursor of the “extrinsic pathway” of coagulation
Factor VIII the antihemophilic factor (hemophilia A) that, after activation, participates catalytically in the activation of factor X. Thrombin cleaves factor VIII to form factor VIIIa
Factor IX precursor of the protease that catalyzes the cleavage of factor X in the “intrinsic pathway” of coagulation
Factor IXa protease catalyzing cleavage of factor X
Factor X precursor of the protease that cleaves prothrombin to produce thrombin
Factor Xa a component of the protease prothrombinase that cleaves prothrombin
Factor XI precursor of the protease that cleaves factor IX to form factor IXa
Factor XIa activates factor IX
Factor XIII precursor to the plasma transglutaminase factor XIIIa that crosslinks fibrin, which prevents its rapid dissociation by dilution from blood flow. Thrombin catalyzes formation of factor XIIIa
Factor XIIIa enzymatically active transglutaminase
F_m_ fibrin monomer
Gene F(*) genes are named similar to the coagulation factors, but substituting the Arabic numeral for the Roman numeral
Gly glycine
FpA fibrinopeptide A
FpB fibrinopeptide B
His histidine
k_c_ catalytic constant of the Michaelis-Menten description of the kinetics of enzyme action
K_m_ Michaelis constant, describing the kinetic constants for the association of substrate with enzyme
Lys lysine
PARs protease-activated receptors
Phe phenylalanine
Pip piperidine
pNA p-nitroaniline
Protein C the vitamin K–dependent protease precursor of the enzyme, protein Ca
Protein Ca Activated protein C or PCa is the inactivation of factors Va and VIIIa
TAFI Thrombin-activated fibrinolysis inhibitor
Tosyl p-toluene sulfonyl
